# Escitalopram versus other antidepressive agents for major depressive disorder: a systematic review and meta-analysis

**DOI:** 10.1186/s12888-023-05382-8

**Published:** 2023-11-24

**Authors:** Juntao Yin, Xiaoyong Song, Chaoyang Wang, Xuhong Lin, Mingsan Miao

**Affiliations:** 1 Department of Pharmacy, Huaihe Hospital, Henan University, Kaifeng, China; 2 National International Cooperation Base of Chinese Medicine, Henan University of Chinese Medicine, Zhengzhou, 450046 China; 3 Department of General Surgery, Huaihe Hospital, Henan University, Kaifeng, China; 4 Department of Clinical Laboratory, Huaihe Hospital, Henan University, Henan, China

**Keywords:** Escitalopram, Antidepressant, Selective serotonin reuptake inhibitors (SSRI), Major depressive disorder (MDD), Meta-analysis

## Abstract

**Background:**

Escitalopram is selective serotonin reuptake inhibitors (SSRIs) and one of the most commonly prescribed newer antidepressants (ADs) worldwide. We aimed to explore the efficacy, acceptability and tolerability of escitalopram in comparison with other ADs in the acute-phase treatment of major depressive disorder (MDD).

**Methods:**

Medline/PubMed, EMBASE, the Cochrane Library, CINAHL, and Clinical Trials.gov were searched from inception to July 10, 2023. Trial databases of drug-approving agencies were hand-searched for published, unpublished and ongoing controlled trials. All randomized controlled trials comparing escitalopram against any other antidepressant for patients with MDD. Responders and remitters to treatment were calculated on an intention-to-treat basis. For dichotomous data, risk ratios (RRs) were calculated with 95% confidence intervals (CI). Continuous data were analyzed using standardized mean differences (with 95% CI) using the random effects model.

**Results:**

A total of 30 studies were included in this meta‑analysis, among which sixteen trials compared escitalopram with another SSRI and 14 compared escitalopram with a newer AD. Escitalopram was shown to be significantly more effective than citalopram in achieving acute response (RR 0.67, 95% CI 0.50—0.87). Escitalopram was also more effective than citalopram in terms of remission (RR 0.53, 95% CI 0.30—0.93).

**Conclusions:**

Escitalopram was superior to other ADs for the acute phase treatment of MDD in terms of efficacy, acceptability and tolerability. However, no significant difference was found between escitalopram and other ADs in early response or follow-up response to treatment of MDD.

**Supplementary Information:**

The online version contains supplementary material available at 10.1186/s12888-023-05382-8.

## Introduction

Major depressive disorder (MDD) is a mood disorder which can lead to a persistent feeling of persistent sadness and loss of interest [1]. The lifetime prevalence of MDD is between 10–20% [2–4]. Recent estimates in 204 countries and territories found that the global prevalence and burden of MDD increased by 27.6% in 2020 due to the COVID-19 pandemic [5]. MDD is the most serious disease in disability-adjusted life years (4.3%), and is estimated to be the leading cause of morbidity worldwide by 2030 if such trend continues [6]. The etiology of MDD is multifactorial, and social, cultural, genomic, aging and other underlying biological factors all play a role [7–12].

Both pharmacological and non-pharmacological treatments are effective for MDD [13], however, antidepressant drugs (ADs) remain the mainstay of treatment in primary and secondary medical institutions [14]. Amongst ADs, there are many different agents are available, including tricyclic antidepressants (TCAs), monoamine oxidase inhibitors (MAOIs), selective serotonin reuptake inhibitors (SSRIs), serotonin-noradrenaline reuptake inhibitors (SNRIs), and other newer ADs. The use of ADs is growing globally, especially in high-income countries [15], mainly due to the increasing consumption of SSRIs and newer ADs [16, 17]. SSRIs are generally better tolerated than TCAs, though the difference in efficacy is slight or negligible [18]. However, head-to-head comparisons have provided inverse findings. Duloxetine, for example, may have the edge over SSRIs in terms of efficacy [19, 20]. In addition, individual SSRIs and SNRIs may have varied outcomes [21]. SSRIs are first-line treatments for MDD [22]; however, these drugs work slowly and, in some patients, may not even work [23]. Escitalopram, one of SSRIs, is the representative of antidepressants currently used in terms of safety and efficacy [20, 23].

Recently, new randomized controlled trials (RCTs) of escitalopram in the treatment of MDD are pouring out, which were conducted in different circumstances [24–26], and the integration effects of these studies was ambiguous. Therefore, it is urgent to determine the true effect size of escitalopram for clinicians and clinical pharmacists. The aim of this present meta-analysis was to evaluate the efficacy of escitalopram in alleviating the acute symptoms of MDD, and to investigate the acceptability and adverse effects (AEs) of escitalopram in comparison with other ADs.

## Methods

This meta-analysis was performed according to the Preferred Reporting Items for Systematic Reviews and Meta-Analyses (PRISMA) guidelines [27]. A review protocol with search strategy was registered in the PROSPERO (CRD42022364229).

### Search strategy

Electronic databases, including Medline/PubMed, EMBASE, the Cochrane Library, CINAHL, and Clinical Trials.gov, were searched to identify relevant studies from inception to July 10, 2023. No restrictions on language, publication status or gender were imposed. The reference lists of all included articles were also manually searched to identify any potential studies that might qualify.

### Selection criteria

Only RCTs were included. Participants who were 18 years or older with a primary diagnosis of MDD were eligible. Studies prior to the 1990s may have used ICD-9, DSM-III/DSM-III-R or other diagnostic criteria. Later studies were more likely to have used criteria of DSM-IV or ICD-10. Studies using Research Diagnostic Criteria or Feighner criteria were included. However, ICD-9 criteria cannot be operationalized, so studies using ICD-9 were excluded. Studies in which no more than 20% of the participants might have bipolar depression were included.

Experimental intervention drug is escitalopram (as monotherapy). Comparator intervention drugs are other ADs for MDD, including TCAs, heterocyclic ADs, SSRIs, and newer ADs (SNRIs, MAOIs, newer agents, and non-conventional ADs such as herbal products). There were no restrictions on dose, frequency, intensity, and duration. Other types of psychotropic agents, such as anxiolytics, anticonvulsants, antipsychotics or mood-stabilizers, were excluded. Depressive patients with severe concomitant diseases, Axis I or II disorders were also excluded. Studies were excluded only if data were not provided at the time of meta-analysis.

The primary outcome was number of participants who responded to treatment, showing a reduction of at least 50% on the Hamilton Depression Scale (HAM-D) or Montgomery-Asberg Depression Rating Scale (MADRS), or any other depression scale, or score less than 2 on CGI-Improvement. The HAM-D was preferred to judge response when more than one criterion was provided. Secondary outcomes included number of participants who achieved remission, scores of change from baseline to the time point in question, acceptability and tolerability. The cut-off point for remission was preset to be score (1) less than 7 on the 17-item HAM-D or less than 8 for all the other longer versions of HAM-D, or (2) less than 12 on the MADRS, or (3) less than 2 on CGI-Severity. The HAM-D was preferred to judge remission when two or more criteria were provided. Change scores from baseline to the time point in question (early response, acute phase response, or follow-up response as defined above) were provided based on HAM-D, MADRS, or any other depression scale. We adopted a looser form of intention-to-treat (ITT) analysis, namely all the participants with more than one post-baseline measurement were represented by their final observations. Acceptability was assessed by total dropout rate, dropout rates due to inefficacy, and dropout rates due to AEs. Tolerability was assessed by total number of patients experiencing at least one AEs, total number of participants experiencing Deaths and suicide. In order to avoid missing any relatively rare yet important AEs, in the data extraction phase, we collected all AEs data reported in the literature and discussed methods for post-hoc summarization.

All titles and abstracts were checked by two reviewers independently (JY and XS) to determine if they met the rough inclusion criteria. All the studies rated as possible candidates by either of the two reviewers were added to the preliminary list. All the full-text articles in the preliminary list were then inspected independently by two reviewers (CW or XL) to determine whether they met the strict inclusion criteria. Any discrepancies were resolved by discussion, or adjudication of a third reviewer (MM).

### Data extraction

Data were collected by two reviewers (JY and CW) using a Microsoft Excel spreadsheet. The extracted data included: author, year of publication, sample size, age, study duration, dose, diagnostic criteria, outcome measures, response criteria, remission criteria rate, overall discontinuation rate, discontinuation rate due to AEs. At the end of the data extraction phase, all key extracted data were reviewed and quality checked by the same two reviewers. Any discrepancies were resolved by discussion at first; and then brought to a third author (MM) for resolution, as required.

### Risk of bias assessment

Two authors (JY and YC) independently used the Cochrane “Risk of bias” (ROB 2.0) tool to assess the methodological quality of the included trials [28].

### Statistical analysis

Data analyses were performed using RevMan 5.4 (Copenhagen: The Nordic Cochrane Centre, The Cochrane Collaboration, 2020). To evaluate heterogeneity, we used the *I*^*2*^ statistic (with *I*^*2*^ > 50% indicating significant heterogeneity) [29] and Cochran’s Q *P* value (with *P* < 0.05 indicating significant heterogeneity). Risk ratio (RR) with 95% confidence interval (CI) was described by categorical data. Standardized mean difference (SMD) with 95% CI was used for continuous outcomes.

Publication bias was evaluated by visual inspection of funnel plot [30]. We performed subgroup analyses to determine whether the results were influenced by the different types of control groups (other SSRIs or newer ADs). Sensitivity analyses were conducted to evaluate the robustness of the synthesized results by excluding studies whose dropout rate was greater than 20%.

## Results

### Study selection and characteristics

**Fig. 1:**
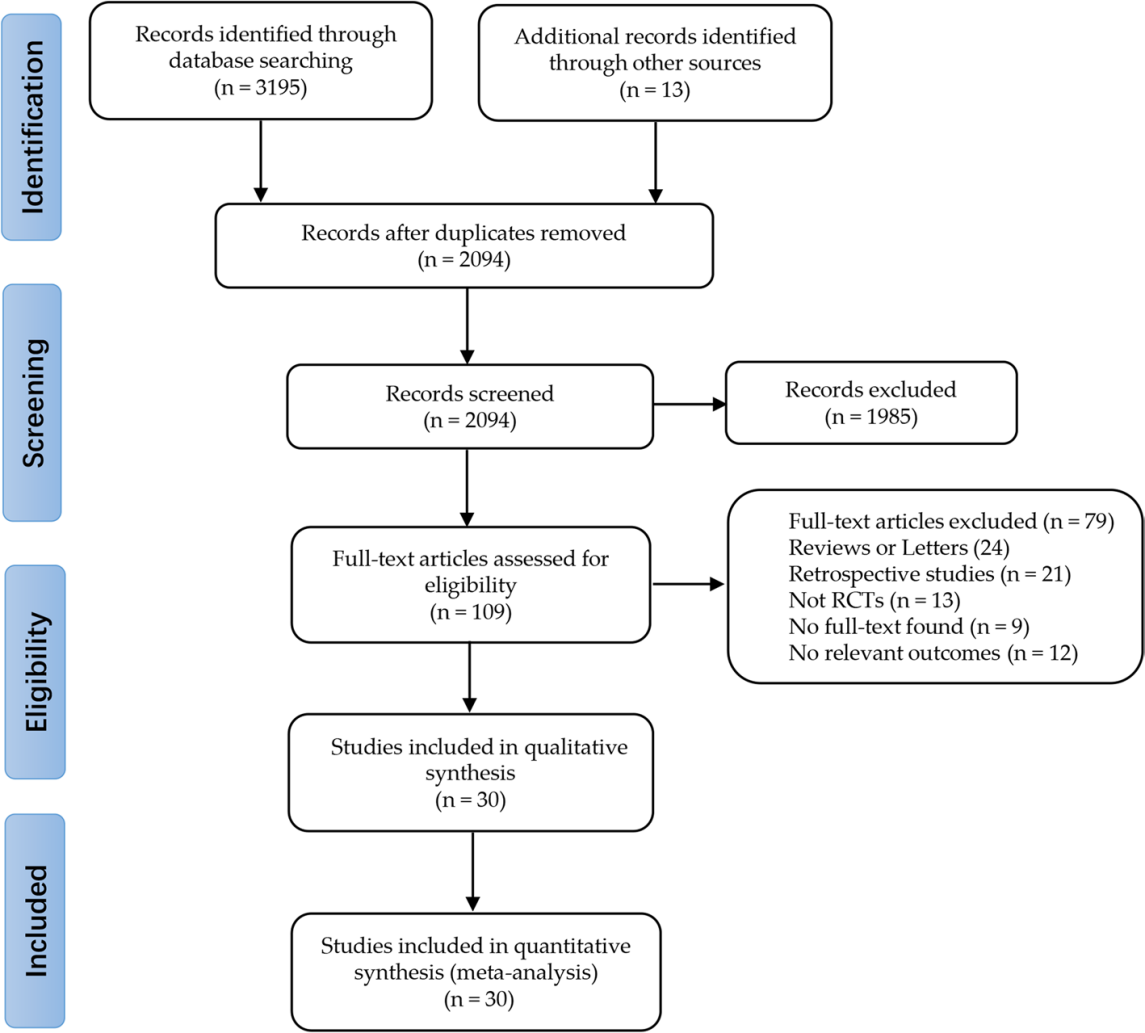
Flowchart of study selection

The preliminary search yielded 109 references of potentially eligible studies. After exclusion of studies that were not relevant (mainly for reviews were or non-randomized studies), a total of 30 RCTs were included in this present review (Fig. 1) [24–26, 31–57].

The basic characteristics for all included studies are displayed in Table 1. In the presentation of the following analyses, a post-hoc decision was made to present all SSRIs (with sub-totals) together in one group, and SNRIs and newer ADs (without sub-totals) together in another group. Sixteen trials (53.3%) compared escitalopram with another SSRI and fourteen (46.7%) compared escitalopram with a newer AD (venlafaxine, bupropion, duloxetine, agomelatine, vilazodone and desvenlafaxine). Neither trials comparing escitalopram with TCAs or MAOIs. Among the total 30 included studies, 28 studies were multicenter, randomized, double-blind trials, the other 2 was randomized, open-label trial. Of all the 30 studies, 17 had an overall high risk of bias, 11 had some concerns of bias, and 2 had a low risk (Figure S1).

**Table 1:** Characteristics of Included Studies

Study	Method	Sample (n) Esc/other ADs	Age (years)	Study duration (weeks)	Drug/dose	Diagnostic criteria	Outcome measures	Response criteria/ response rate Esc: other ADs	Remission criteria/remission rate	Overall discontinuation rate Esc: other ADs
Corruble et al. 2013 [39]	RCT	160/164	18–70	12	Esc/10-20 mg/day, Agomelatine/25-50 mg/day	DSM-IV-TR, HAMD, CGI-S	HAMD, CGI-I, CGI-S	80.0%/83.2%	54.4%/60.9%	20/164/23/160
Udristoiu et al. 2016 [26]	RCT	143/144	18–65	12	Esc/10–20 mg/day, Agomelatine/25–50 mg/day	DSM-IV-TR, QIDS-SR16, HAMD, CGI-S, SDS	QIDS-SR16, HAMD-17, CGI-I, CGI-S, SDS	68.4%/62.9%	-	22/143/21/144
Clayton (AK130926) [31]	RCT	149/138	≥ 18	8	Esc/10–20 mg/day, Bupropion XR/300–450 mg/day	DSM-IV for HAMD	HAMD	82/149/81/138	56/149/54/138	44/149/39/138
Clayton (AK130927) [32]	RCT	138/141	≥ 18	8	Esc/10–20 mg/day, Bupropion XR/300–450 mg/day	DSM-IV for HAMD	HAMD	90/138/82/141	65/138/54/141	33/138/32/141
SCT-MD-35	RCT	138/147	18–80	8	Esc/4 mg/day, Bupropion XR/150 mg/day	DSM-IV for MADRS	MADRS, HAMD	56/138/51/138	35/138/34/138	32/138/34/138
Soares et al. 2010 [53]	RCT	299/296	40–70	8	Esc/10–20 mg/day, Desvenlafaxine/100–200 mg/day	MADRS, HAMD	HAMD-17, CGI-S	73%/64%	48%/38%	43/299/51/296
Khan et al. 2007 [43]	RCT	140/138	18–80	8	Esc/10–20 mg/day, Duloxetine/60 mg/day	DSM-IV for MADRS	MADRS	92/140/63/138	60/140/48/138	21/140/46/138
Nierenberg et al. 2007 [48]	RCT	274/273	≥ 18	8	Esc/10 mg/day, Duloxetine/60 mg/day	DSM-IV for MADRS, CGI-S	HAMD	109/274/113/273	85/274/97/273	66/274/85/273
Wade et al. 2007 [55]	RCT	144/151	18–65	24	Esc/10 mg/day, Duloxetine/60 mg/day	MADRS, HAMD, CGI-S	MADRS, HAMD, CGI-I, CGI-S	115/144/112/151	94/144/87/151	32/144/37/151
Bielski et al. 2004 [35]	RCT	101/101	18–65	8	Esc/20 mg/day, Venlafaxine XR/225 mg/day	DSM-IV for MADRS	MADRS, HDRS, CGI-I, CGI-S	57/101/47/101	40/101/36/101	29/101/35/101
Montgomery et al. 2004 [46]	RCT	148/145	18–85	8	Esc/10–20 mg/day, Venlafaxine XR/75–150 mg/day	DSM-IV for MADRS	MADRS, HAMD, CGI-I, CGI-S	113/148/113/145	102/148/99/145	23/148/21/145
Kadam et al. 2020 [41]	RCT	16/17	18–60	12	Esc/20 mg/day, Vilazodone/20 mg	HAMD-17, MADRS	HAMD-17, MADRS	-	-	0/16/0/17
Kudyar et al. 2018 [25]	RCT	26/24	18–55	6	Esc/10 mg/day, Vilazodone/20 mg/day	DSM	HAMD	-	-	0/26/0/24
Kumar et al. 2023 [57]	RCT	26/26	18–55	4	Esc/10–20 mg/day, Vilazodone/20–40 mg	HDRS, MADRS, CGI-S	HDRS, MADRS			2/26/6/26
Burke et al. 2002 [37]	RCT	252/127	18–65	8	Esc/10–20 mg/day, Citalopram/40 mg/day	DSM-IV for MADRS	MADRS, HDRS, CGI-I, CGI-S	122/252/57/127	77/252/34/127	63/252/34/127
Colonna et al. 2005 [38]	RCT	175/182	18–65	24	Esc/10 mg/day, Citalopram/20 mg/day	DSM-IV for MADRS	MADRS, CGI-I, CGI-S	132/175/136/182	-	31/175/47/182
Lepola et al. 2003 [44]	RCT	156/161	18–65	8	Esc/10–20 mg/day, Citalopram/20–40 mg/day	DSM-IV for MADRS		95/156/79/161	78/156/63/161	10/156/9/161
Moore et al. 2005 [47]	RCT	142/152	18–65	8	Esc/20 mg/day, Citalopram/40 mg/day	DSM-IV for MADRS	MADRS	105/142/87/152	77/142/62/152	10/142/25/152
SCT-MD-02	RCT	129/128	18–80	8	Esc/10–20 mg/day, Citalopram/20–40 mg/day	DSM-IV for MADRS, HAMD	MADRS, HAMD, CGI-I, CGI-S	55/129/54/128	39/129/39/128	33/129/29/128
Yevtushenko et al. 2007 [56]	RCT	109/110	25–45	6	Esc/10 mg/day, Citalopram/20 mg/day	DSM-IV for MADRS	MADRS	103/109/90/110	97/109/55/110	1/109/2/110
Ou et al. 2011 [49]	RCT	115/117	18–65	6	Esc/10–20 mg/day, Citalopram/20–40 mg/day	DSM-IV-TR, HAMD-17	HAMD-17	72.17% (83/115)/74.36% (87/117)	60.87%(70/115)/56.41%(66/117)	0/115/0/117
Kasper et al. 2005 [42]	RCT	174/164	≥ 65	8	Esc/10 mg/day, Fluoxetine/20 mg/day	DSM-IV for MADRS	MADRS	79/174/61/164	68/174/50/164	30/174/47/182
Kennedy et al. 2005 [40]	RCT	102/103	18–80	8	Esc/10–20 mg/day, Fluoxetine/20–40 mg/day	DSM-IV for MADRS	MADRS, HAMD, CGI-I, CGI-S	66/102/63/103	48/102/48/103	36/102/26/103
Mao et al. 2008 [45]	RCT	123/117	18–65	8	Esc/10 mg/day, Fluoxetine/20 mg/day	CGI-S, DSM-IV for MADRS	MADRS	94/123/89/117	64/123/62/117	15/123/17/117
SCT-MD-09	RCT	16/14	18–55	5	Esc/10–20 mg/day, Fluoxetine/20–40 mg/day	DSM-IV for MADRS, HAMD	MADRS, HAMD	-	-	1/16/2/14
Baldwin et al. 2006 [34]	RCT	166/159	22–40	8	Esc/10–20 mg/day, Paroxetine/20–40 mg/day	MADRS	MADRS, HDRS, CGI-I, CGI-S	112/166/111/159	93/166/97/159	15/166/14/159
Boulenger et al. 2006 [36]	RCT	232/227	18–75	24	Esc/20 mg/day, Paroxetine/40 mg/day	DSM-IV for MADRS	MADRS, HDRS, CGI-I, CGI-S	188/232/171/227	-	47/232/74/227
Kishi et al. 2017 [24]	RCT	43/45	20–70	24	Esc/5–20 mg/day, Paroxetine/12.5–50 mg/day	HAMD-17	HAMD-21, HAMD-17	32/43/27/45	23/43/20/45	24/43/27/45
Alexopoulos et al. 2004 [33]	RCT	136/138	18–65	8	Esc/10–20 mg/day, Sertraline/50–200 mg/day	DSM-IV for MADRS	MADRS, HDRS, CGI-I, CGI-S	68/136/76/138	48/136/56/138	28/136/25/138
Ventura et al. 2007 [54]	RCT	107/108	18–80	8	Esc/10 mg/day, Sertraline/ 50–200 mg/day	DSM-IV for MADRS	MADRS, HAMD, CGI-I, CGI-S	78/107/75/108	60/107/62/108	19/107/15/108

*CGI-I* Clinical Global Impressions-Severity of Illness, *CGI-S* Clinical Global Impressions-Improvement scales, *DSM* Diagnostic and Statistical Manual of Mental Disorders, *Esc* Escitalopram, *HAMD* Hamilton Rating Scale for Depression, *MADRS* Montgomery-Asberg Depression Rating Scale, *RCT* Randomised controlled trial

The effects of interventions in efficacy, acceptability and tolerability are presented below. The results are reported by comparison (dividing SSRIs from newer ADs). AEs are only reported when statistically significant.

### Number of patients who responded to treatment

#### Acute phase treatment (6 to 12 weeks)

**Fig. 2:**
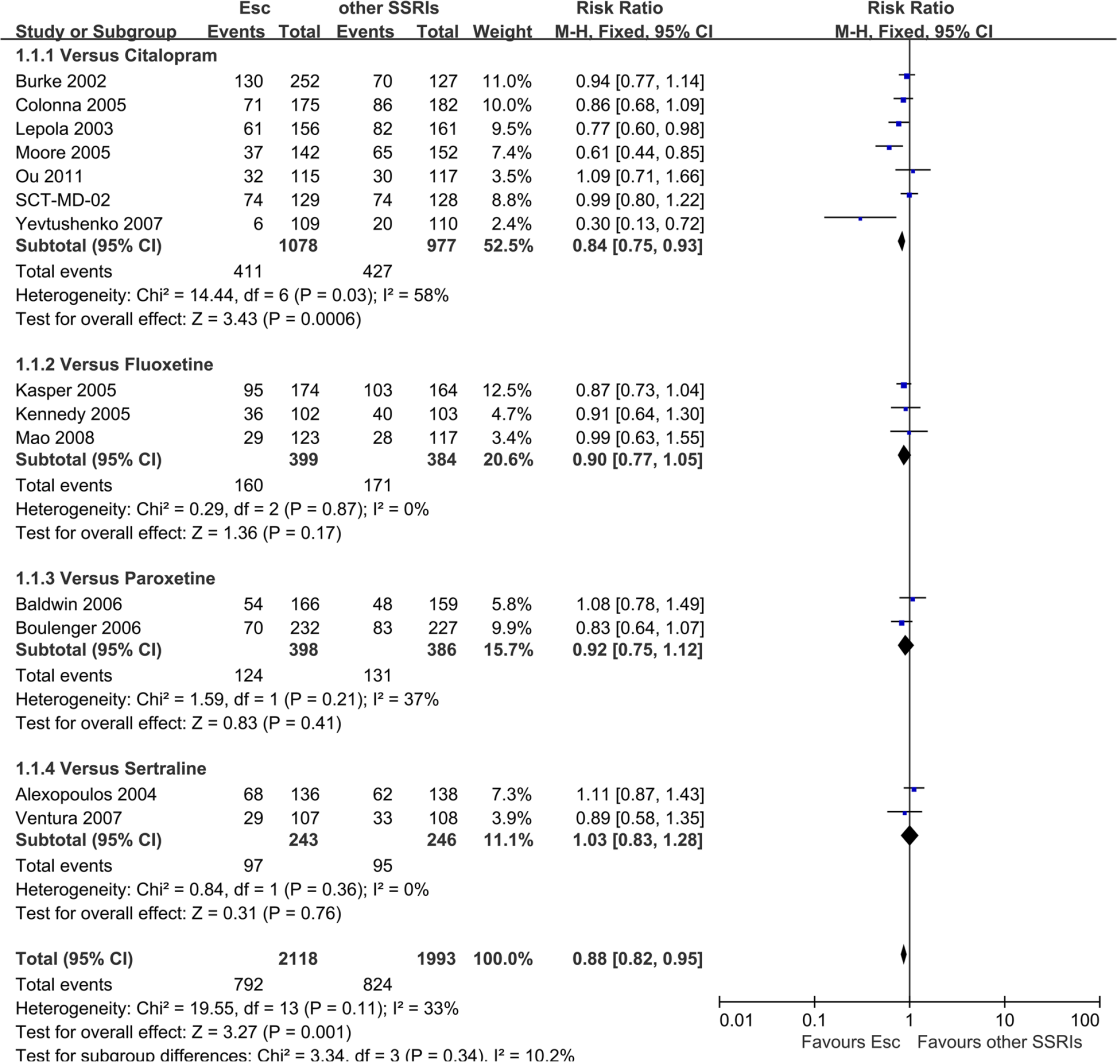
Failure to respond at endpoint (6–12 weeks): Escitalopram versus other SSRIs

**Fig. 3:**
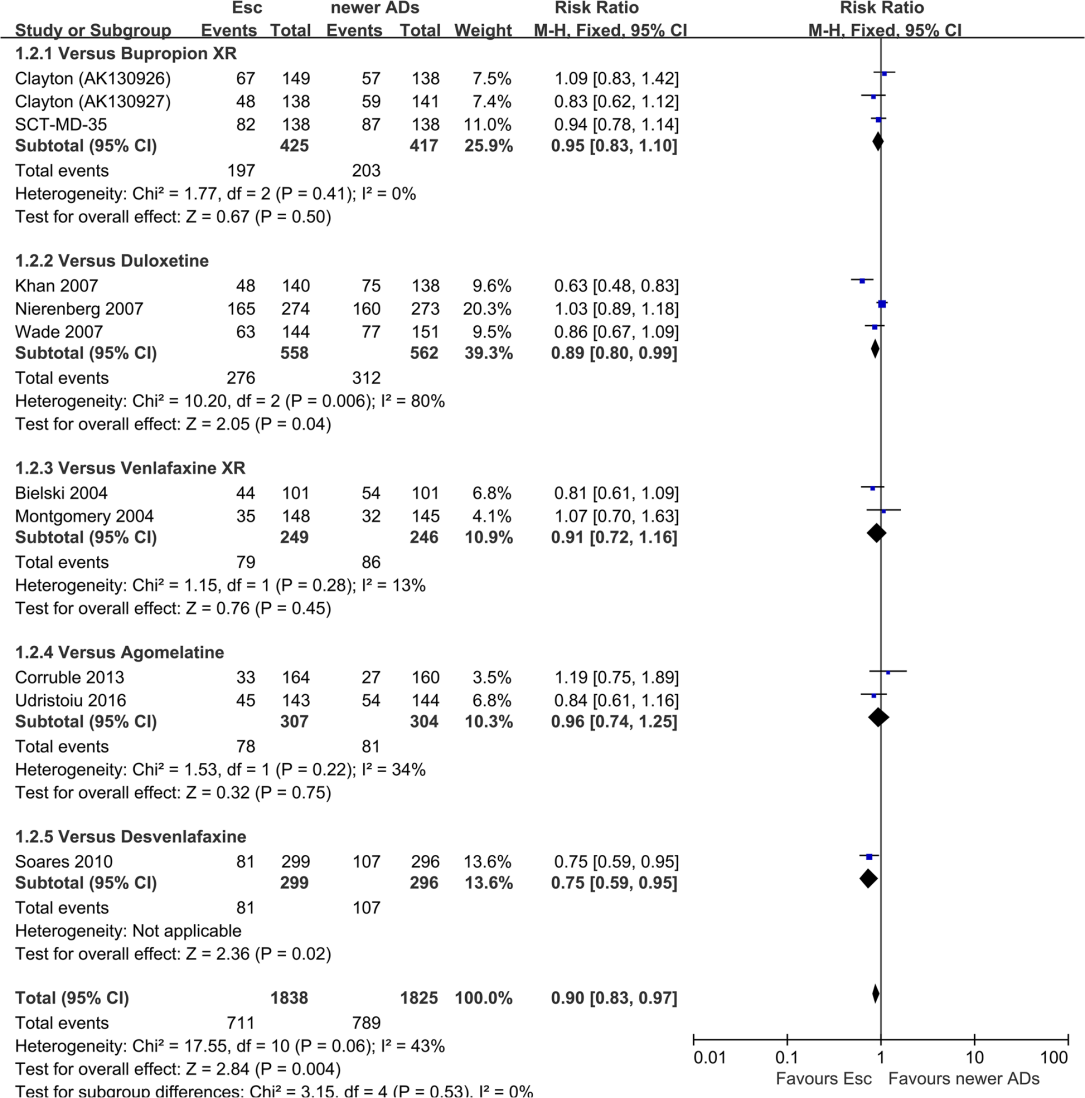
Failure to respond at endpoint (6–12 weeks): Escitalopram versus newer ADs

There was a statistically significant difference with escitalopram being more effective than other SSRIs (RR 0.88, 95% CI 0.82 to 0.95, *I*^*2*^ = 33%; 14 studies, 4111 participants) (Fig. 2). There was a statistically significant difference with escitalopram being more effective than newer ADs (RR 0.90, 95% CI 0.83 to 0.97, *I*^*2*^ = 43%; 11 studies, 3663 participants) (Fig. 3).

#### Early response (1 to 4 weeks)

There was no statistically significant difference with escitalopram being more effective than other SSRIs (RR 1.02, 95% CI 0.93 to 1.11) (Figure S2) or newer ADs (RR 0.97, 95% CI 0.87 to 1.08) (Figure S3).

#### Follow-up response (16 to 24 weeks)

There was no statistically significant difference between escitalopram and other SSRIs (RR 0.83, 95% CI 0.66 to 1.05, *I*^*2*^ = 0%) (Figure S4). And there was no statistically significant difference between escitalopram and newer ADs (RR 0.78, 95% CI 0.51 to 1.19) (Figure S5).

### Number of patients who achieved remission

#### Acute phase treatment (6 to 12 weeks)

**Fig. 4:**
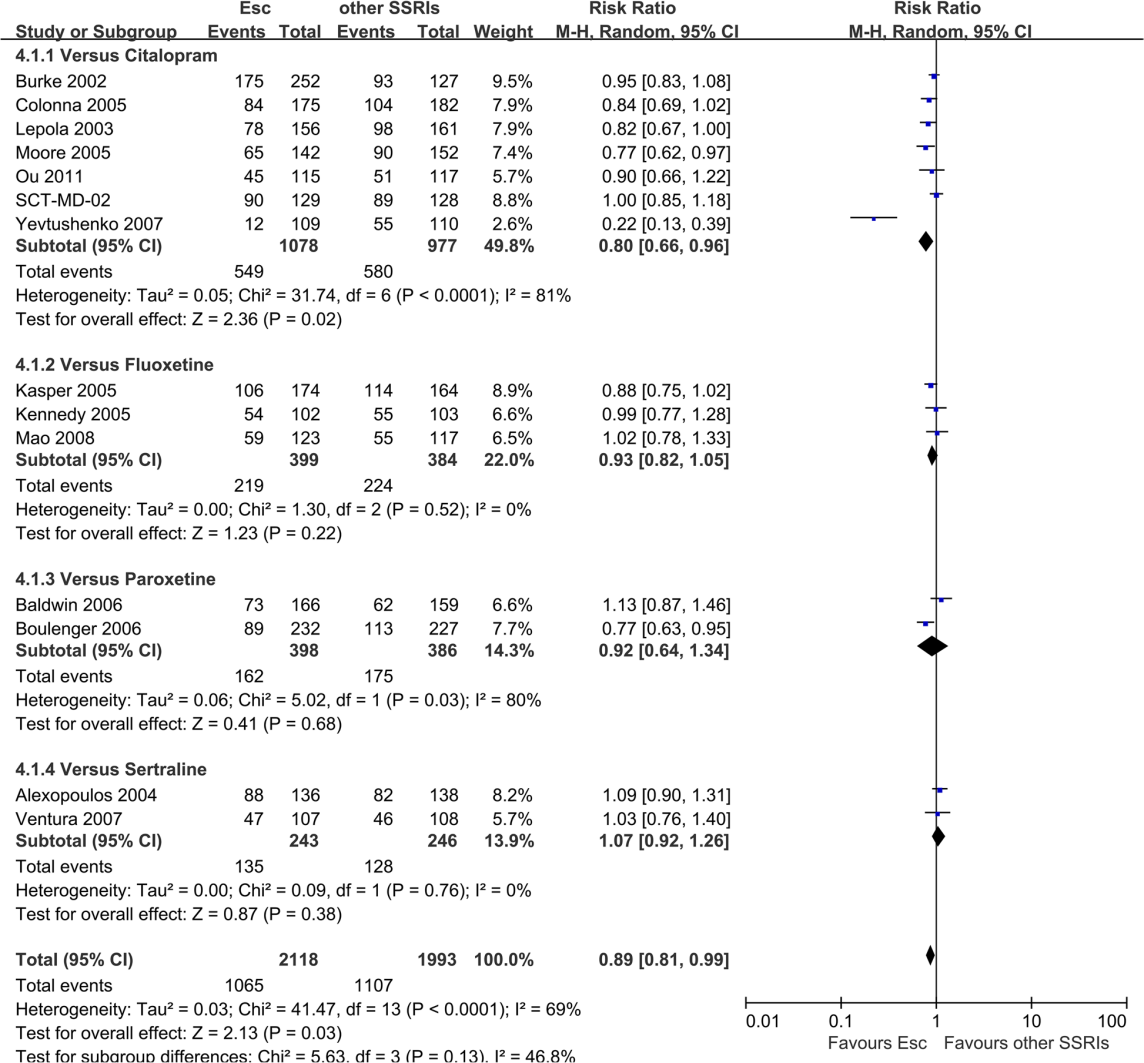
Failure to remission at endpoint (6–12 weeks): Escitalopram versus other SSRIs

There was statistically significant difference between escitalopram being more effective than other SSRIs (RR 0.89, 95% CI 0.81 to 0.99, *I*^*2*^ = 69%) (Fig. 4), however, there was no statistically significant difference with escitalopram being more effective than newer ADs (RR 0.96, 95% CI 0.91 to 1.01, *I*^*2*^ = 36%) (Figure S6).

#### Follow-up remission (16 to 24 weeks)

There was no statistically significant difference between escitalopram being more effective than other SSRIs (RR 0.84, 95% CI 0.55 to 1.27) (Figure S7) or newer ADs (RR 0.82, 95% CI 0.61 to 1.10) (Figure S8).

#### Mean change from baseline (6 to 12 weeks)

Escitalopram was found to be more efficacious than other SSRIs in reduction of depressive symptoms (SMD -0.13, 95% CI -0.19 to -0.06, *I*^*2*^ = 34%) (Figure S9) or newer ADs (SMD -0.41, 95% CI -0.81 to -0.02, *I*^*2*^ = 97%) (Figure S10).

### Tolerability-Total number of patients experiencing at least one side effect

**Fig. 5:**
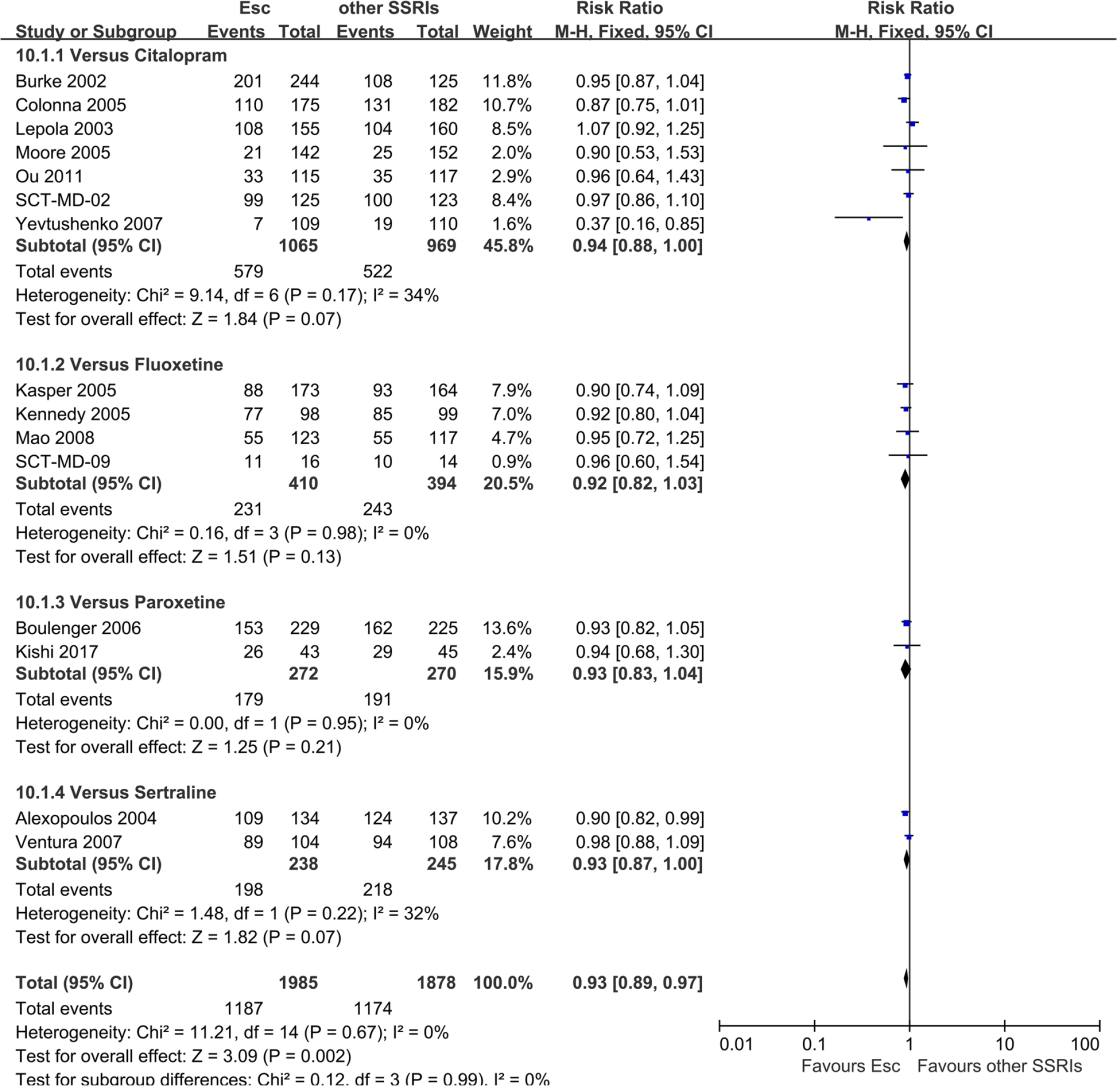
Subjects with at least one TEAE: Escitalopram versus other SSRIs

There were statistically significant differences between escitalopram and other SSRIs in terms of tolerability (RR 0.93, 95% CI 0.89 to 0.97, *I*^*2*^ = 0%) (Fig. 5). However, there were no statistically significant differences between escitalopram and newer ADs in terms of tolerability (RR 0.97, 95% CI 0.93 to 1.01, *I*^*2*^ = 29%) (Figure S11).

### Sensitivity analysis

The trials whose dropout rates were greater than 20% were excluded in the sensitivity analysis. Referring to other SSRIs, a dropout rate greater than 20% was found for three studies comparing escitalopram with citalopram [37, 49, 50], one with fluoxetine [40] and one with paroxetine [36]. Among newer ADs, a dropout rate greater than 20% was found for all the three studies comparing escitalopram with bupropion [31, 32, 52], two with duloxetine [48, 55], two with agomelatine [26, 39], one with desvenlafaxine [53], one with vilazodone [25], and one with venlafaxine [35]. Three studies had only one arm reporting a dropout rate greater than 20% [38, 42, 43]. Therefore, sensitivity analysis was conducted only for the comparisons between escitalopram and other SSRIs.

Results from the sensitivity analyses remained in favor of escitalopram, not only when studies whose dropout rate was greater than 20% in both arms were ruled out (RR 0.81, 95% CI 0.68 to 0.97, *I*^*2*^ = 68%; 7 studies, 1893 participants; Figure S12), but also when studies whose dropout rate was greater than 20% in only one arm were additionally ruled out (RR 0.71, 95% CI 0.51 to 0.98, *I*^*2*^ = 71%; 4 studies, 1062 participants; Figure S13). Therefore, results from these sensitivity analyses did not materially change the main findings, suggesting that the pooled analyses were robust.

### Publication bias

Funnel plot of the studies enrolled in the meta-analysis demonstrated no significant asymmetry by visual inspection, therefore, the outcome of failure to respond at 6–12 weeks (escitalopram *vs*. other SSRIs) was not affected by publication bias (Figure S14).

## Discussion

Thirty studies were included in this review. Escitalopram was superior to other SSRIs or newer ADs for the acute phase treatment of MDD in terms of efficacy (citalopram, fluoxetine, desvenlafaxine and duloxetine) and acceptability (Paroxetine and duloxetine). A quarter of the included trials used citalopram as the comparator and only a few trials per comparison were found for most of the remaining ADs (with the exception of duloxetine, fluoxetine, paroxetine and bupropion), which limits the power of this study to detect moderate but clinically meaningful differences between the drugs. The randomized evidence collected in the datasets for this review was sufficient to detect differences in early response to treatment (after two weeks of intervention). However, checking the data reported in the studies included in this meta-analysis, the question on comparative efficacy of early onset response has not been resolved and remains a controversial issue.

To the best of our knowledge, our work is the most comprehensive review to date of the comparative efficacy, acceptability and tolerability of escitalopram and other ADs for the treatment of MDD. The sample size in our study was larger than that of similar systematic reviews comparing an AD versus other ADs [58, 59]. Escitalopram is a relatively new compound and the quality of psychiatric trials may have improved over the past few years. By using a broader scope and advanced statistical methods, our findings provide more certainty about results than previous reviews which assessed similar research questions [20, 60]. Although these studies also focused on RCTs to assess the comparative safety and efficacy of drugs, we employed meta-analyses which enabled us to use a more comprehensive evidence base including head-to-head trials.

It has long been argued that placebo-controlled trials are required to adequately demonstrate the efficacy of newer ADs [61], however, receiving placebo in RCTs increased the chances of dropout and decreased the absolute response of participants to active ADs [62]. In the case of ADs, it may be more appropriate to conduct trials using an active comparator (chosen from the most effective and better-tolerated treatments available) [63]. Therefore, in this review, we included only head-to-head trials comparing between escitalopram and other active treatments. Because the literature search was comprehensive, it might be impossible that some studies had not been identified.

Our study demonstrated that escitalopram was superior to other ADs for the acute phase treatment of MDD in terms of efficacy, acceptability and tolerability. The mechanism action of escitalopram to improve MDD especially in the acute phase may be characterized by the increased subcortical network-ventral attention network connectivity [64], lower plasma kynurenine levels and resting-state regional activity in the left dorsolateral prefrontal cortex [65].

There are some limitations in this review. At First, although the sample size was larger, most studies still do not report adequate information on randomization and allocation concealment. For example, outcomes that were clearly relevant to patients and clinicians, in particular, patients' and their caregivers' attitudes to interventions, their ability to resume work and normal social functioning, were not reported in the enrolled studies. Furthermore, information on randomization and allocation concealment was occasionally lacking, which may be due to reporting in the text than real defects in study design. At last, the reports of the outcomes in the included studies were often unclear or incomplete and the figures used for the analyses were not easy to understand. And sometimes there were some inconsistencies between published data and unpublished data on the websites of pharmaceutical industries. In order to make up for these limitations, we evaluated the risk of bias in the results of trials, and preferred to report meta-analyses restricted to trials with low risk of bias [66–68].

In terms of AEs profile, we found that different ADs showed different tolerability profiles, which is an important issue from a clinical point of view, and the outcomes of this study are consistent with previous findings [69, 70]. However, a full description of tolerability profile of drugs cannot rely solely on randomized evidence [71, 72]. In addition, AEs were inconsistently reported in the included studies in our review, which hampers cross-study comparisons. The reporting of AEs needs to be standardized, and more consideration should be given to patients' subjective experience of medication. During the evidence-based decision-making process, clinicians should consider and inform patients of different AEs profiles among ADs, therefore, the issue on tolerability is clinically important. However, it has been shown that RCTs might not be the most effective tool for identifying possible causal relationship between ADs and even severe adverse events (SAEs) [73]. This applies to class-related AE, but might also apply to each specific compound. The more information that is pooled together in a systematic review and meta-analysis, the more precise and accurate is the estimate [74]. We are also aware of the possibility that a number of RCTs comparing escitalopram with other ADs are currently underway [75]. With more reliable and longer-term studies, the real impact and burden of the newer ADs on treated patients in terms of tolerability will be known.

Moreover, clinicians should take dosage and duration into consideration when administering drug therapy. In this review, nearly all studies used dosages and durations within the therapeutic range. Sixteen studies used a flexible-dose regimen and the remaining fourteen used a fixed-dose one. Among the included studies, there was no evidence of imbalance in terms of dosage, duration, or disease severity in favor of the investigational drug.

## Conclusion

Our study demonstrated that escitalopram appears to be suitable as first-line antidepressant treatment for moderate to severe MDD. Escitalopram was superior to other ADs for the acute phase treatment of MDD in terms of efficacy, acceptability and tolerability. However, there is insufficient evidence to detect a difference between escitalopram and other ADs in early response or follow-up response to treatment of MDD. Sponsorship bias may lead to overestimation of treatment effects; therefore, results reported for comparative efficacy should be treated with caution.

### Supplementary Information

**Additional file 1: Figure S1. **Risk of bias summary. **Figure S2. **Failure to respond (at 1-4 weeks): Escitalopram versus other SSRIs. **Figure S3. **Failure to respond (at 1-4 weeks): Escitalopram versus newer ADs. **Figure S4. **Failure to respond (at 16-24 weeks): Escitalopram versus other SSRIs. **Figure S5. **Failure to respond (at 16-24 weeks): Escitalopram versus newer ADs. **Figure S6. **Failure to remission at endpoint (6-12 weeks): Escitalopram versus newer ADs. **Figure S7. **Failure to remission (at 16-24 weeks): Escitalopram versus other SSRIs. **Figure S8. **Failure to remission (at 16-24 weeks): Escitalopram versus newer ADs. **Figure S9. **Standardized mean difference at endpoint (6-12 weeks): Escitalopram versus other SSRIs. **Figure S10. **Standardized mean difference at endpoint (6-12 weeks): Escitalopram versus newer ADs. **Figure S11. **Subjects with at least one TEAE: Escitalopram versus newer ADs. **Figure S12. **Excluding trials whose dropout rate was greater than 20%: Escitalopram versus other SSRIs (dropout rate greater than 20% in both arms). **Figure S13. **Excluding trials whose dropout rate was greater than 20%: Escitalopram versus other SSRIs (dropout rate greater than 20% in only one arm). **Figure S14. **Funnel plot of comparison: Failure to respond at endpoint (6-12 weeks): Escitalopram versus other SSRIs. 

## Data Availability

The datasets used and/or analyzed during the current study are available from the corresponding author upon reasonable request.

## Abbreviations

AE: Adverse effect
AD: Antidepressant
CI: Confidence interval
HAM-D: Hamilton Depression Scale
ITT: Intention-to-treat
MDD: Major depressive disorder
MAOI: Monoamine oxidase inhibitor
MADRS: Montgomery-Asberg Depression Rating Scale
PRISMA: Preferred Reporting Items for Systematic Reviews and Meta-Analyses
RCT: Randomized controlled trial
ROB: Risk of bias
RR: Risk ratio
SSRI: Selective serotonin reuptake inhibitor
SNRI: Serotonin-noradrenaline reuptake inhibitor
SAE: Severe adverse event
SMD: Standardized mean difference
TCA: Tricyclic antidepressant
