# Supplementary material for: Escitalopram versus other antidepressive agents for major depressive disorder: a systematic review and meta-analysis
Source: BMC Psychiatry. 2023 Nov 24;23:876. doi: 10.1186/s12888-023-05382-8 (PMC10675869; doi:10.1186/s12888-023-05382-8)
Supplement: Supplementary file 1 — Additional file 1: Figure S1. Risk of bias summary. Figure S2. Failure to respond (at 1-4 weeks): Escitalopram versus other SSRIs. Figure S3. Failure to respond (at 1-4 weeks): Escitalopram versus newer ADs. Figure S4. Failure to respond (at 16-24 weeks): Escitalopram versus other SSRIs. Figure S5. Failure to respond (at 16-24 weeks): Escitalopram versus newer ADs. Figure S6. Failure to remission at endpoint (6-12 weeks): Escitalopram versus newer ADs. Figure S7. Failure to remission (at 16-24 weeks): Escitalopram versus other SSRIs. Figure S8. Failure to remission (at 16-24 weeks): Escitalopram versus newer ADs. Figure S9. Standardized mean difference at endpoint (6-12 weeks): Escitalopram versus other SSRIs. Figure S10. Standardized mean difference at endpoint (6-12 weeks): Escitalopram versus newer ADs. Figure S11. Subjects with at least one TEAE: Escitalopram versus newer ADs. Figure S12. Excluding trials whose dropout rate was greater than 20%: Escitalopram versus other SSRIs (dropout rate greater than 20% in both arms). Figure S13. Excluding trials whose dropout rate was greater than 20%: Escitalopram versus other SSRIs (dropout rate greater than 20% in only one arm). Figure S14. Funnel plot of comparison: Failure to respond at endpoint (6-12 weeks): Escitalopram versus other SSRIs. [file 12888_2023_5382_MOESM1_ESM.doc]

**Supplementary file**


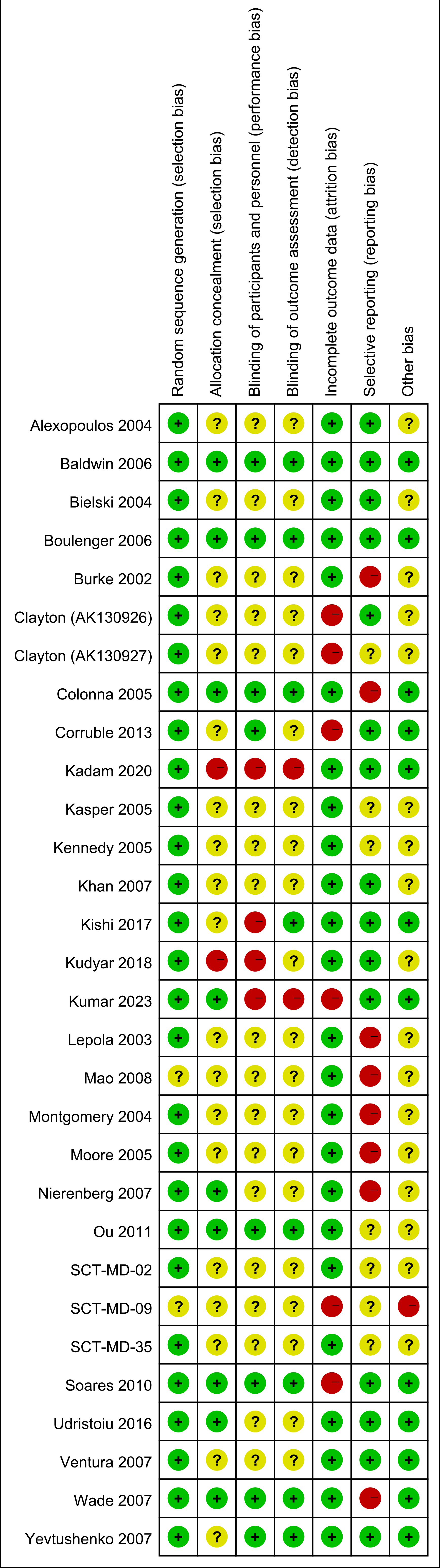


**Figure S1** Risk of bias summary.

**
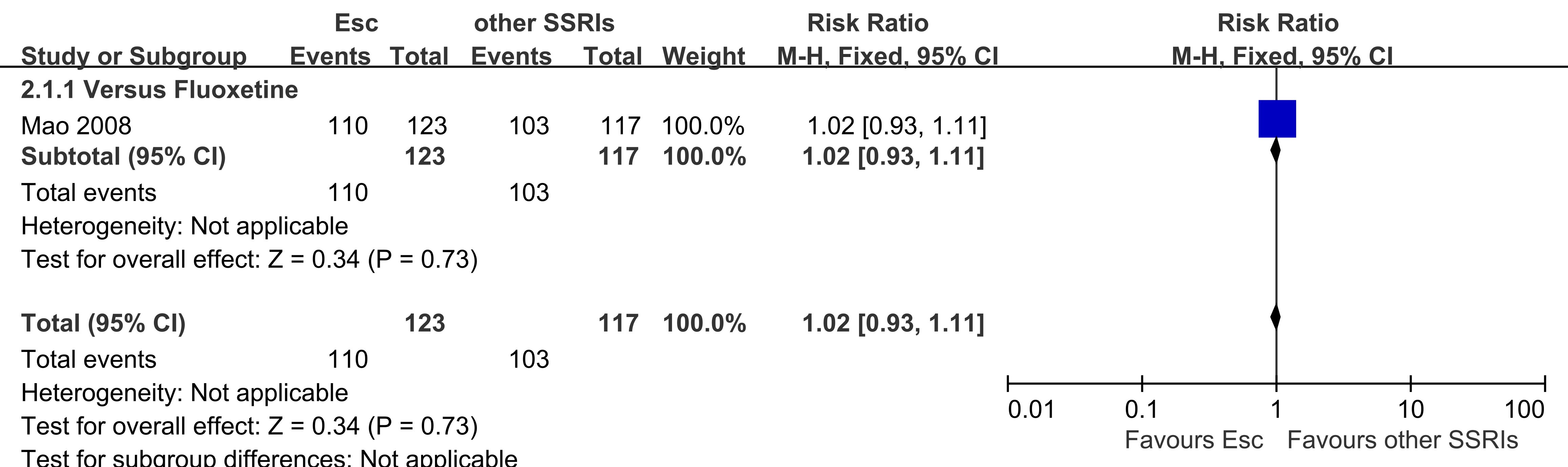
**

**Figure S2** Failure to respond (at 1-4 weeks): Escitalopram versus other SSRIs.

**
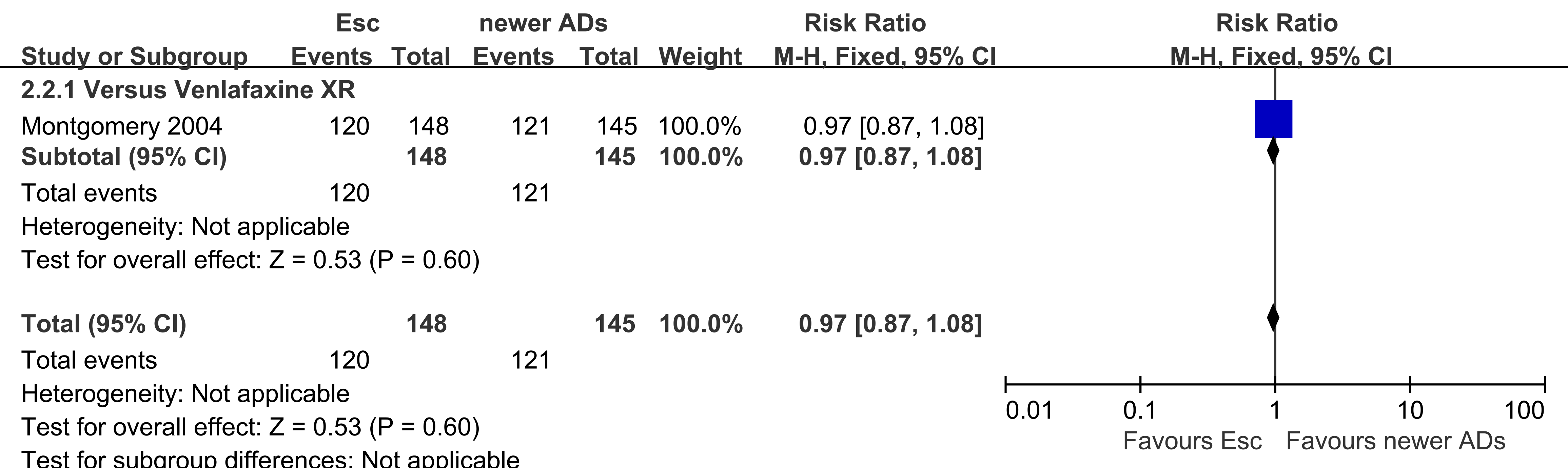
**

**Figure S3** Failure to respond (at 1-4 weeks): Escitalopram versus newer ADs.

**
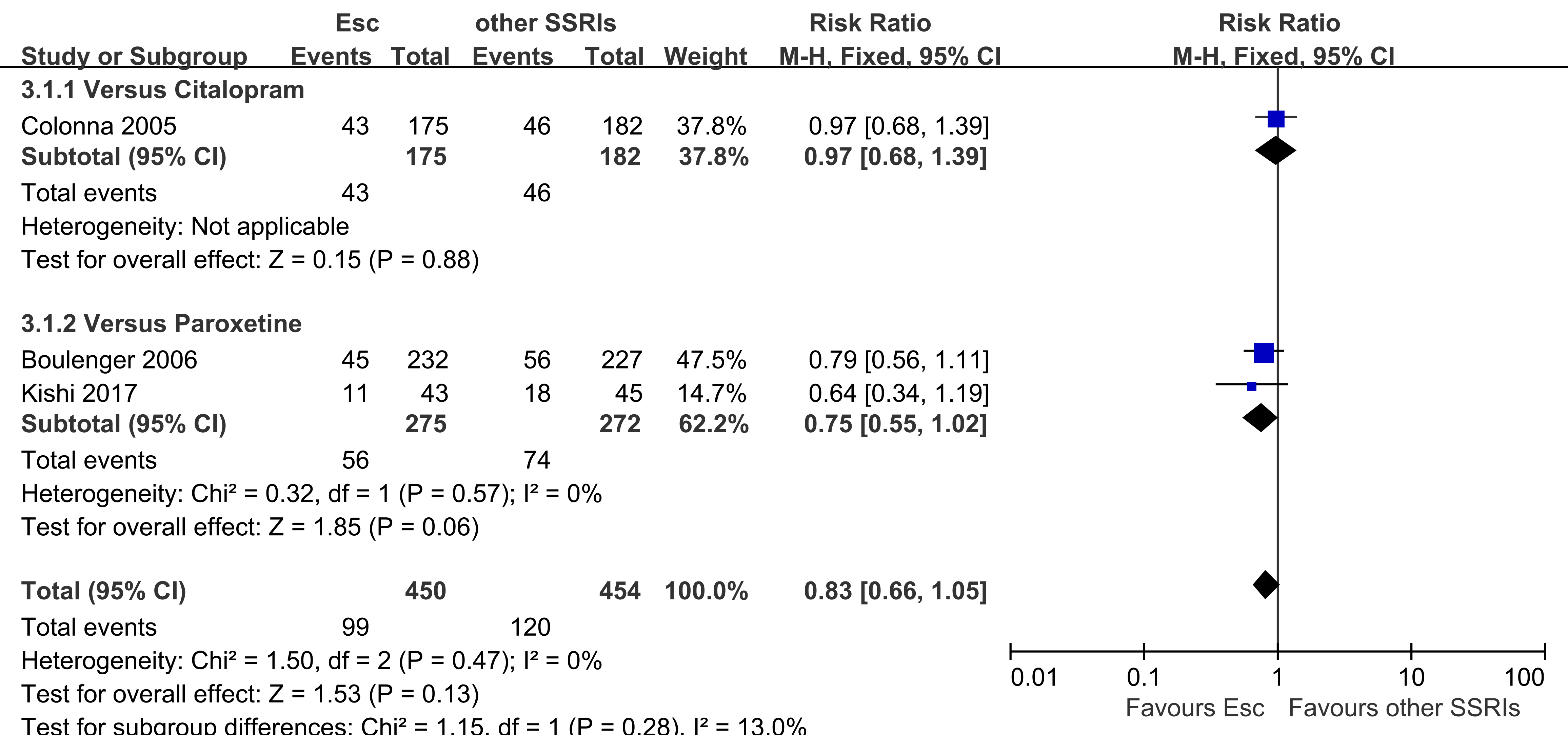
**

**Figure S4** Failure to respond (at 16-24 weeks): Escitalopram versus other SSRIs.

**
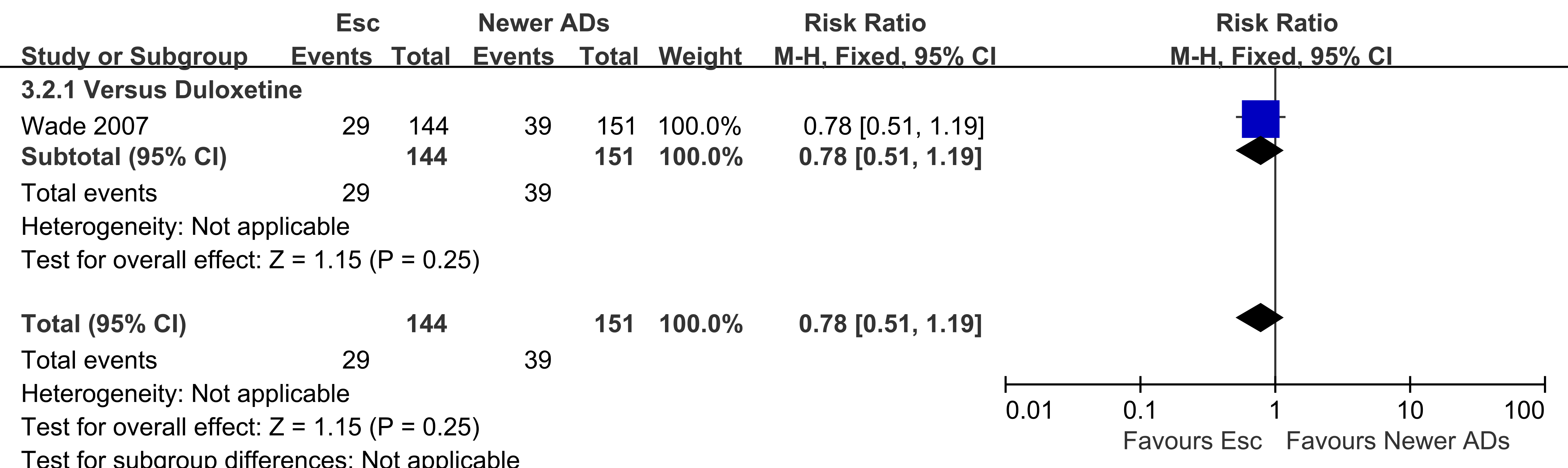
**

**Figure S5** Failure to respond (at 16-24 weeks): Escitalopram versus newer ADs.

**
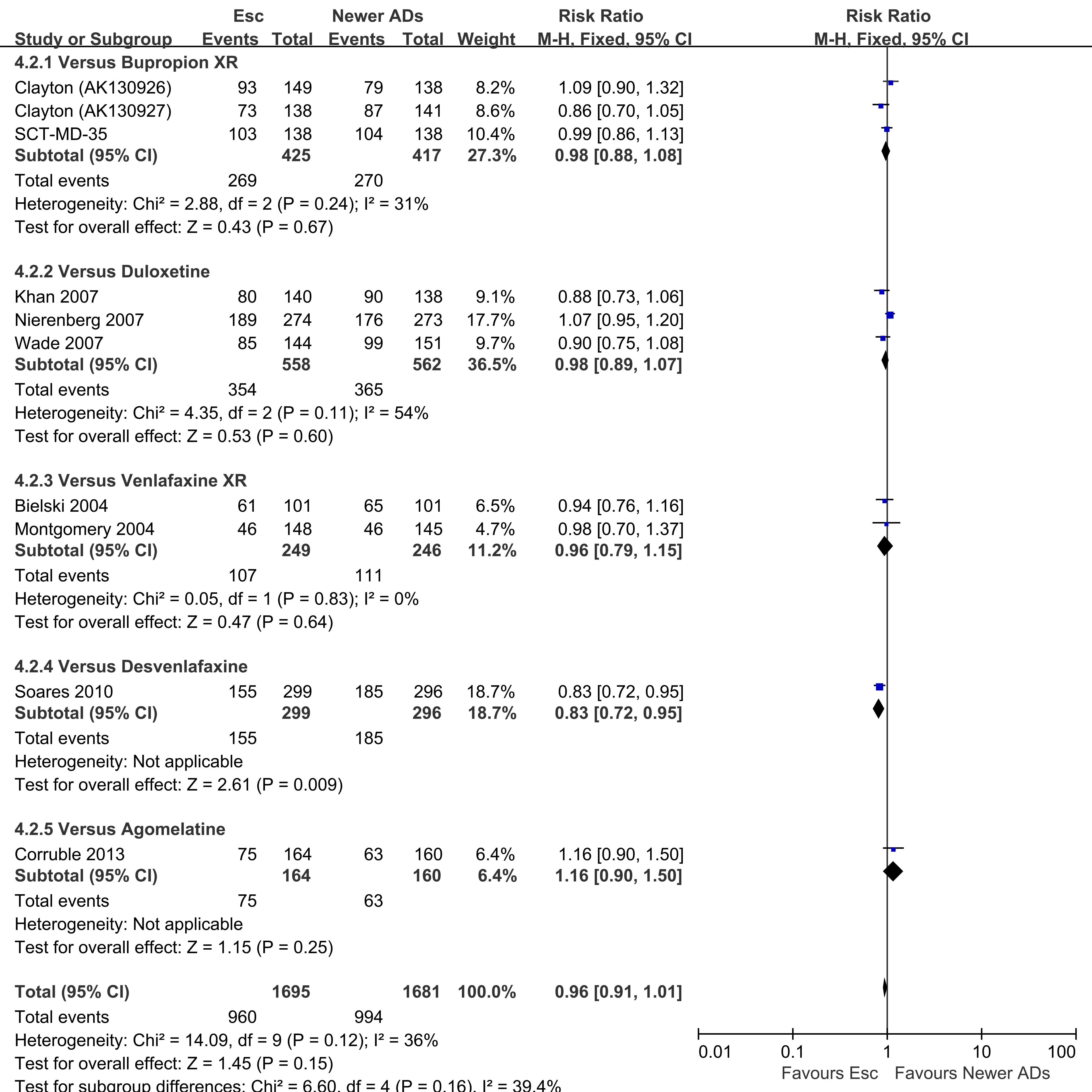
**

**Figure S6** Failure to remission at endpoint (6-12 weeks): Escitalopram versus newer ADs.

**
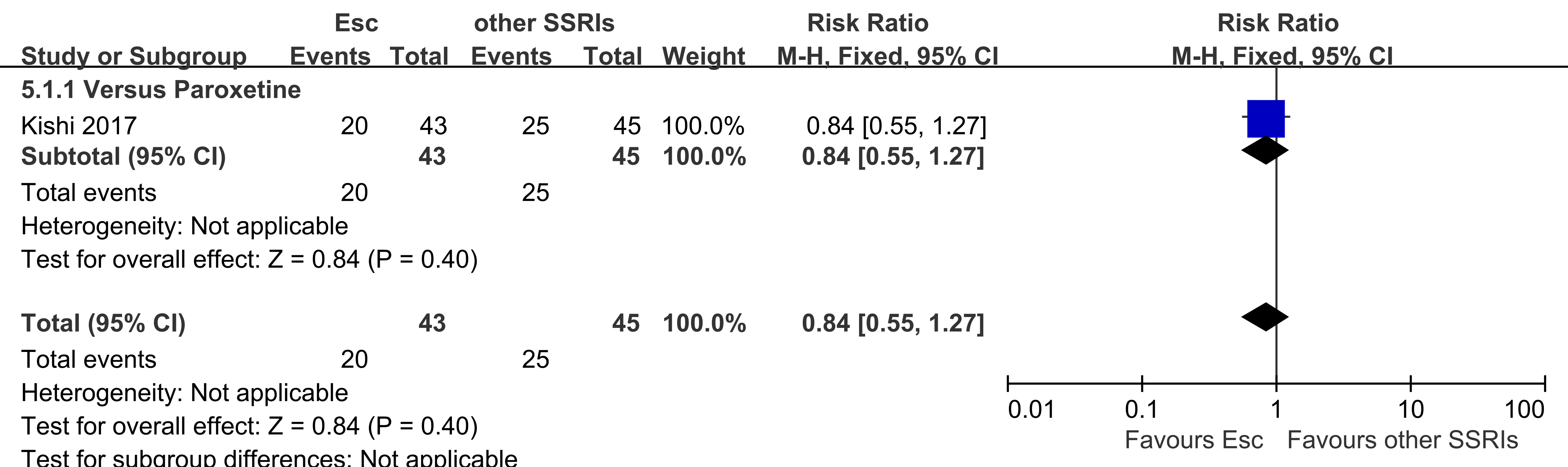
**

**Figure S7** Failure to remission (at 16-24 weeks): Escitalopram versus other SSRIs.

**
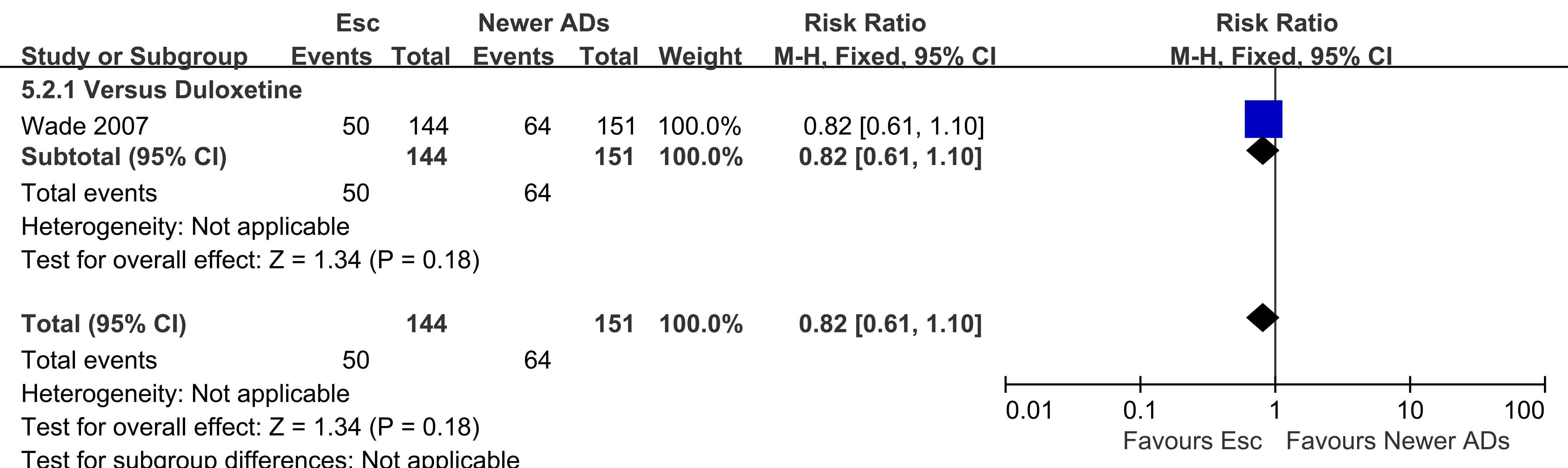
**

**Figure S8** Failure to remission (at 16-24 weeks): Escitalopram versus newer ADs.

**
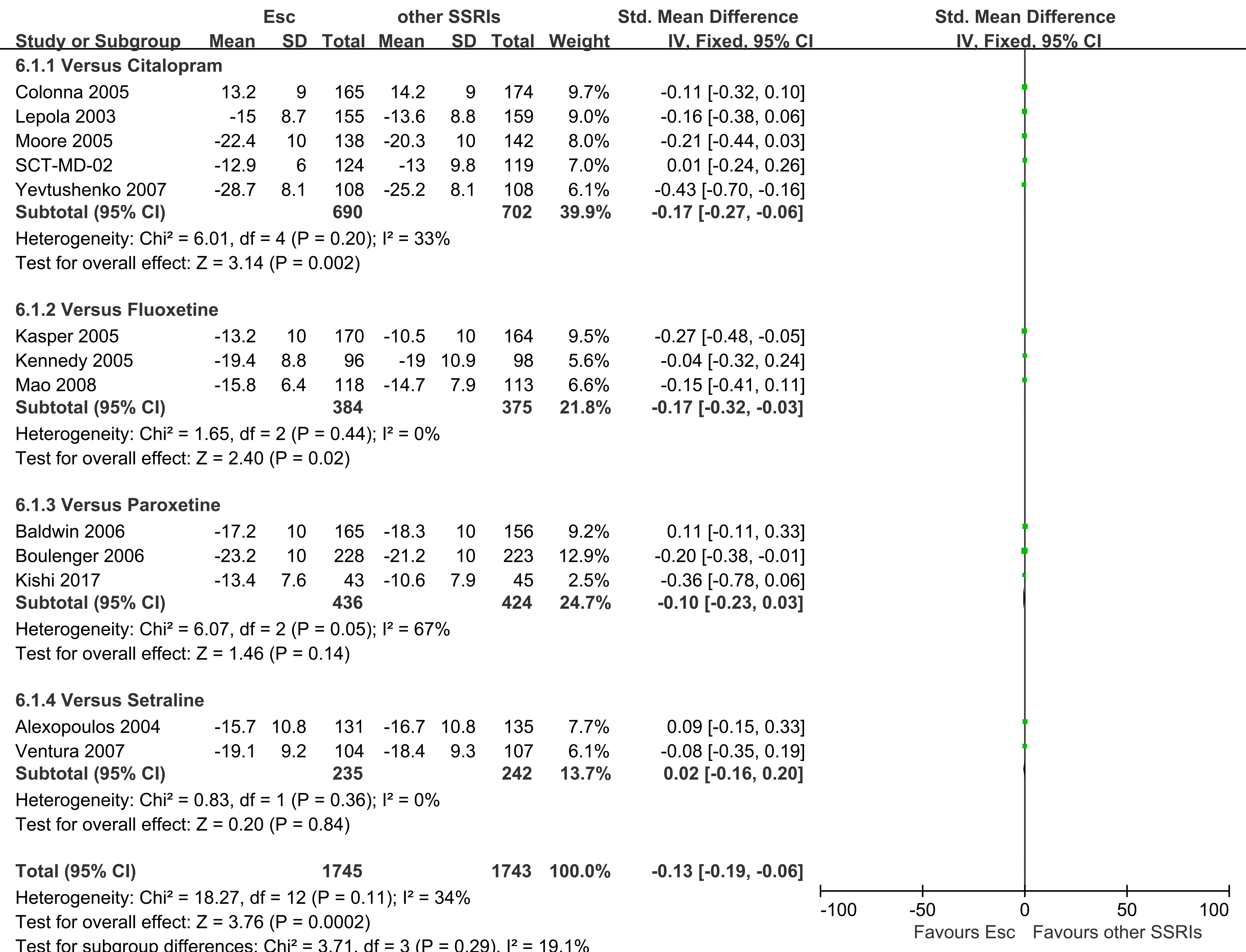
**

**Figure S9** Standardized mean difference at endpoint (6-12 weeks): Escitalopram versus other SSRIs.

**
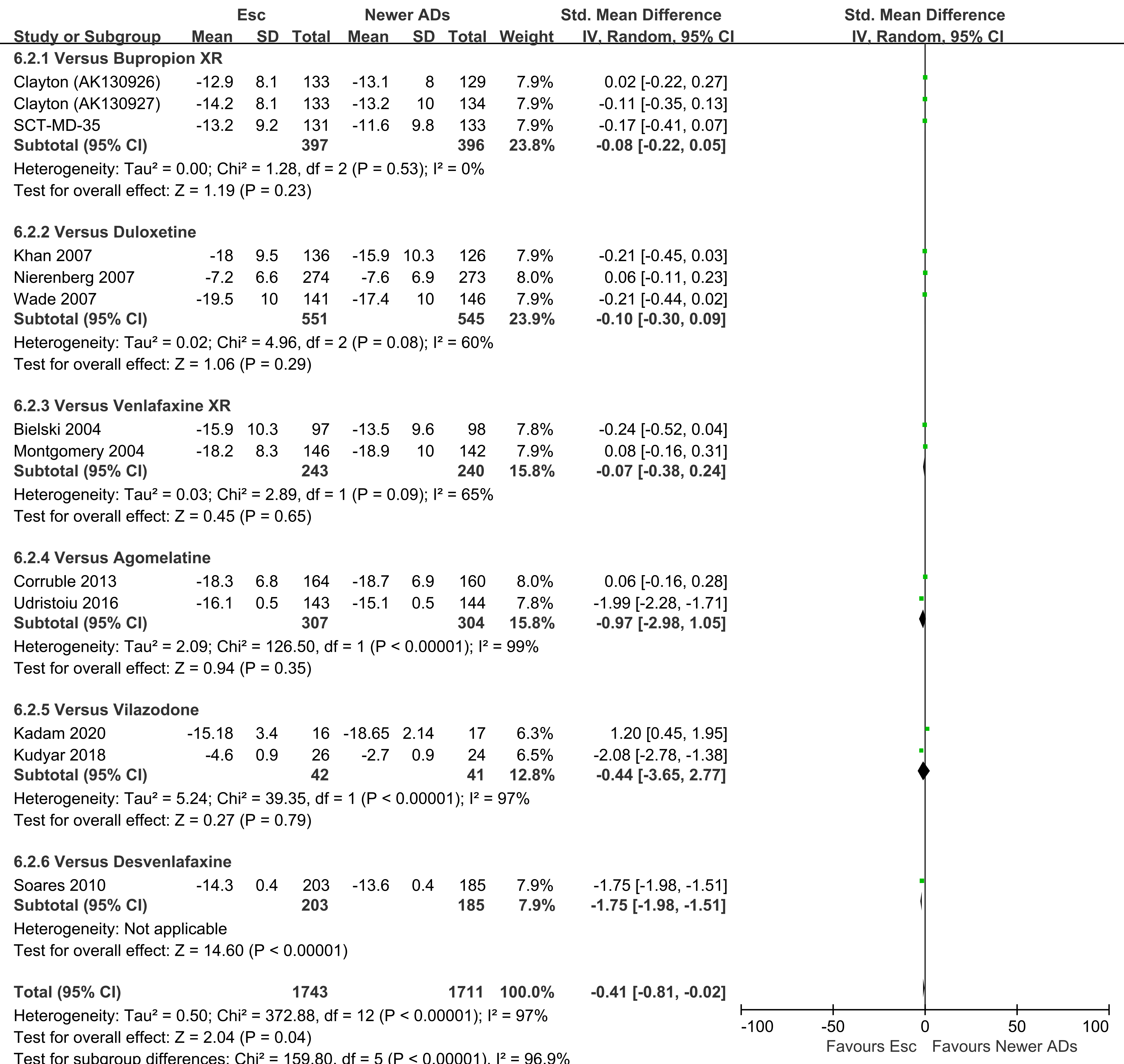
**

**Figure S10** Standardized mean difference at endpoint (6-12 weeks): Escitalopram versus newer ADs.

**
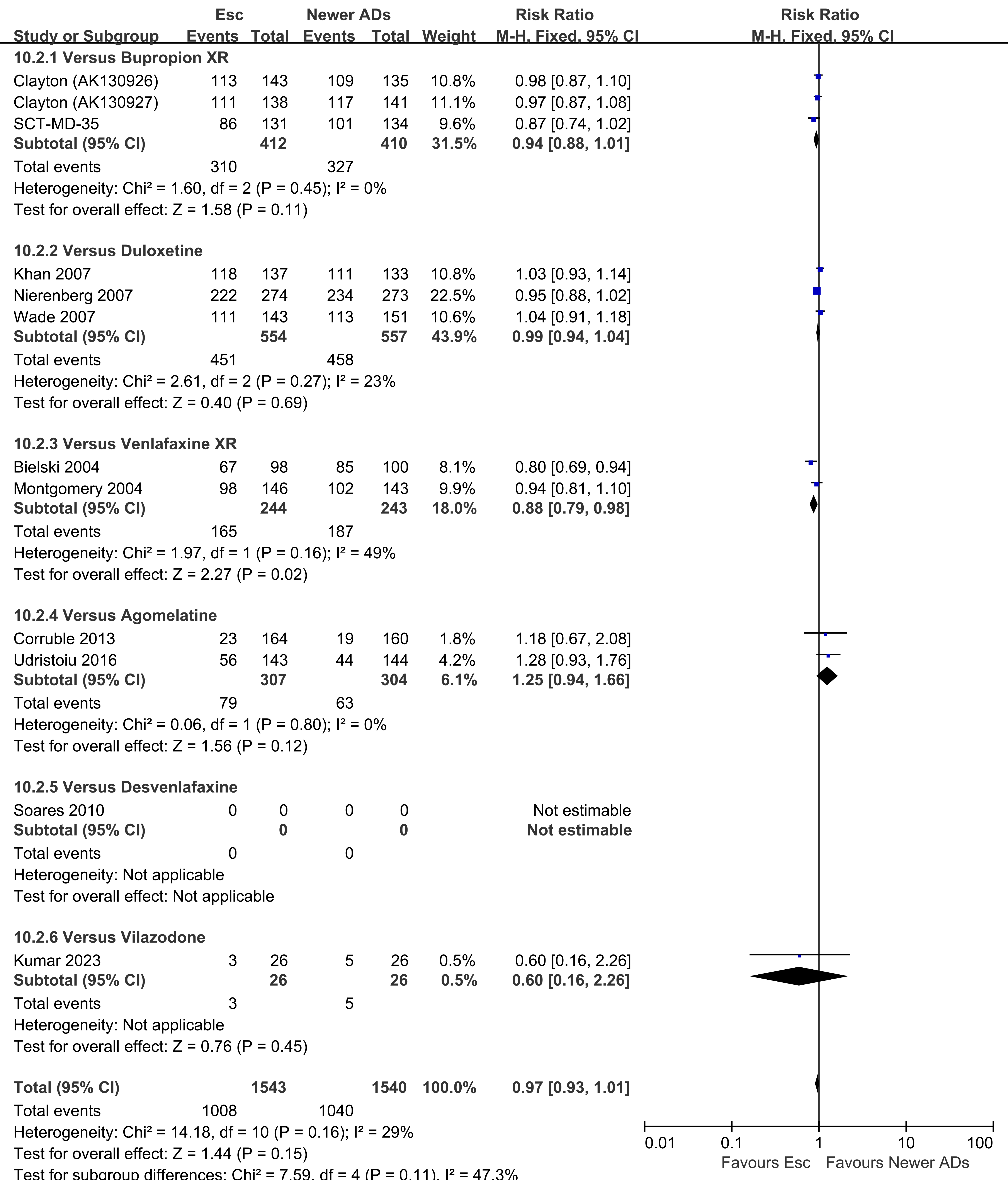
**

**Figure S11** Subjects with at least one TEAE: Escitalopram versus newer ADs.

**
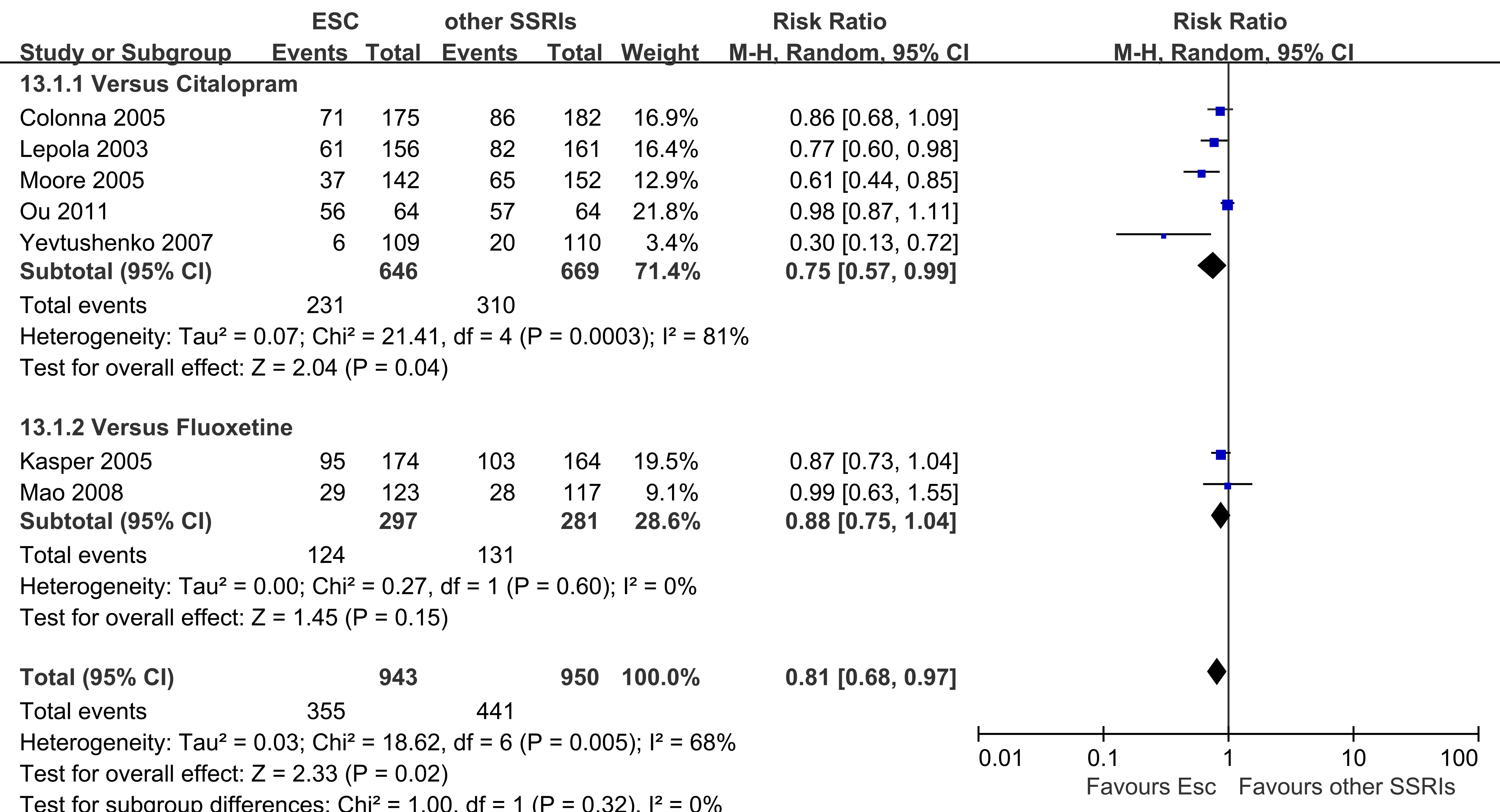
**

**Figure S12** Excluding trials whose dropout rate was greater than 20%: Escitalopram versus other SSRIs (dropout rate greater than 20% in both arms).

**
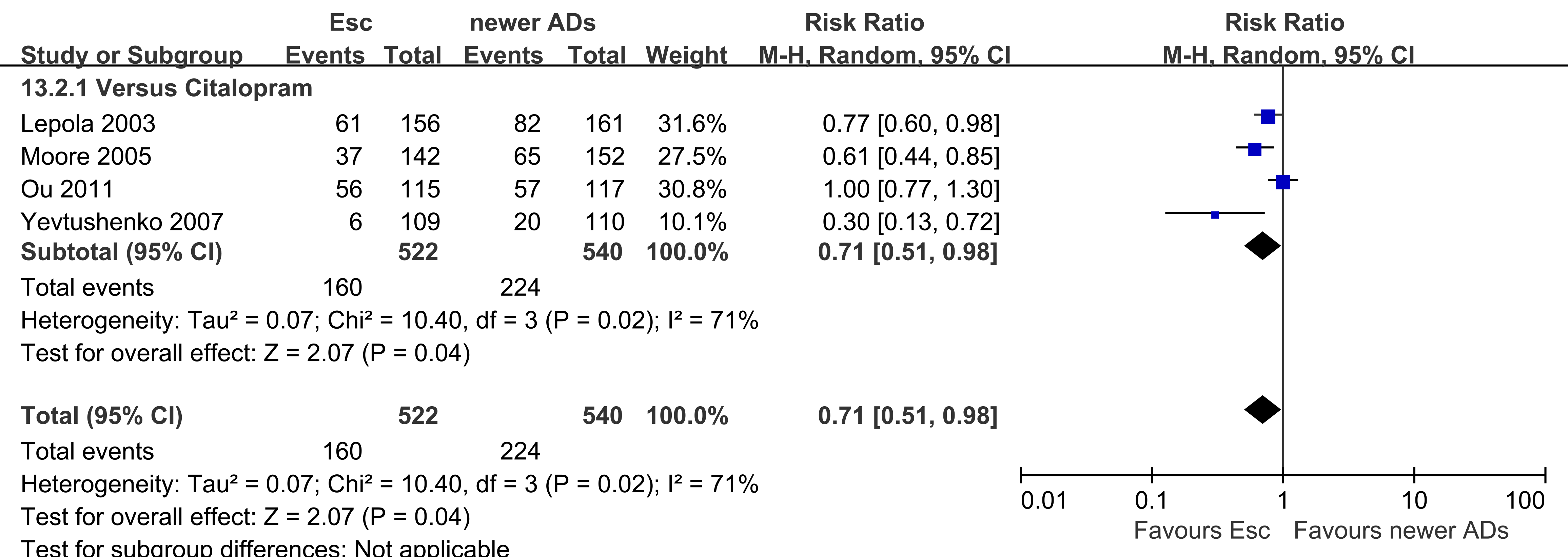
**

**Figure S13** Excluding trials whose dropout rate was greater than 20%: Escitalopram versus other SSRIs (dropout rate greater than 20% in only one arm).

**
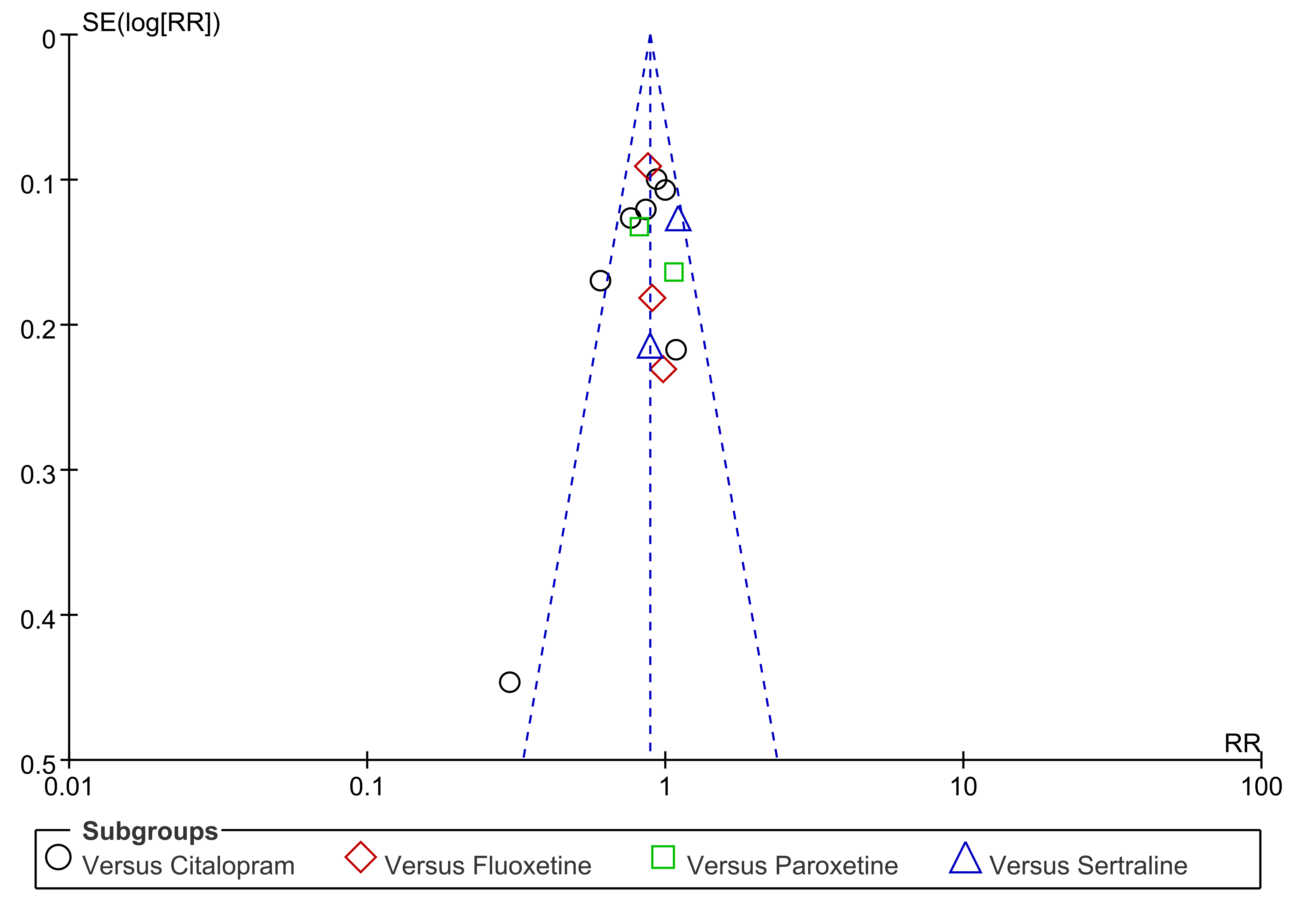
**

**Figure S14** Funnel plot of comparison: Failure to respond at endpoint (6-12 weeks): Escitalopram versus other SSRIs.
